# Dysmenorrhœa

**Published:** 1875-02

**Authors:** John M. Johnson

**Affiliations:** Atlanta, Ga.


ATLANTA

MEDICAL AND SURGICAL JOURNAL.

VOL. XII.] FEBRUARY—1875. [NO. 11.

### Original Communications.

### DYSMENORRHŒA.

BY JOHN M. JOHNSON, M.D., OF ATLANTA, GA.

*Read before the Atlanta Academy of Medicine.*

Neither our philosophy, the logic of speculation, nor the inquiries of physiologists and gynæcologists, have led us to harmonious conclusions regarding the essential nature of menstruation. We have not been able to penetrate the arcanum in which its secrets are hid. The field is an inviting one. Messenger after messenger has gone forth to explore it, and all have returned empty-handed, declaring that its secret springs are not ready to be revealed, and its laws too mystical for the present state of human knowledge. What wonder, then, that we hear the cry from one, Lo, here! and from another, Lo, there!—that one should say it is due to ovarian influence, and another that it is an inherent and independent function; and still another, that it is a co-relative function, requiring the co-operation of other organs to complete it? We should be charitable in all matters of opinion towards each other. We should neither reflect at random, nor condemn at a venture, but, like Paul, believe all things, hope all things, taking care to hold fast to that which is good.

Before proceeding with the subject, I wish to lay down some general principles which will render my views more intelligible. Every organ of the body is independent of every other organ in the essential function assigned to it. The brain and cord, liver and spleen, ovaries and womb, all perform different functions in the living economy. In making a pin, for instance, one man prepares the metal, another makes the wire, another cuts it the proper length, another polishes and sharpens it, another makes the head, and another puts it on. As in mechanics, so with the complete organism; rudimentary nutrition begins in the mouth; the food, masticated and insalivated there, is made homogeneous in the stomach, where certain products are absorbed; it then passes to the duodenum, where a process awaits it; the residium with the fats, after receiving the pancreatic juice and bile, is emulsified; and while in the small bowel, the products appropriate to their functions are absorbed by the lacteals, converted into lymph, and carried into the circulation near the heart by its own duct.

I shall speak of reflex action as a psycho-physiological function, such as occurs in the sexual coitus, in the nutritive processes, and all the animal functions, leaving out, of course, the morbid phenomena. For instance, the hen lays an egg perfect in all its parts, but, if she has not seen the male, the nucleus is wanting, and the physiological action fails. But, having seen the male, she selects her nest in a quiet, hidden place; this is a physical function. She lays an egg in it every day until the nest is full; this is a pyscho-physiological function. She sits on them until they hatch; this is a mixed function, and the hatching is the consummation of the primary embryo-physiological action.

In the hen, ovulation is independent of sexual interference; the egg matures, and is thrown out perfect in every particular, but will not reproduce. In the woman, the same state of facts exists. The egg is formed simultaneously with all the other organs, and is a part of the complete organism, but remains passive until the psycho-physiological forces begin to develop. These consist—first, in a love of offspring; second, the innate desire of every female to be a mother. Gradually the pubes and mammary glands give evidence of approaching puberty. Here begins a new train of psycho-physiological actions. The womb, that has been passive up to this time, now asserts its great function; "pleasure waves her enchanting wand;" erethism begins, the reflex function follows, and the train of physiological actions we call menstruation begins.

The vascularity of the womb is in proportion to the wonderful function it is intended to perform. It is penetrated by innumerable arteries. It is made of lateral and transverse fibres, very small, but very powerful, and held together by equally powerful connective tissue. It is ramified by millions of arterioles and capillaries, only separated from the cavity by a thin epithelium. A stassis is set up: this results in rupturing the epithelium, and relieving the congestion by a flow of blood, which we call menstruation. Another great function marks this period. I mean ovulation. The ovaries are glands. They are analogues of all the other glands. Each has power to organize and mature the products peculiar to it, independently of all interference from other organs. All of these products are utilized by the economic forces. The ovaries, like other glands, contribute their product in the form of a small cell, which is the result of its inherent power. This cell burrows its way through the substance of the gland by an ulcerative process, and falls into the oviduct which transmits it to the womb. Here, when impregnated, it begins the work of organization. Instinct with life, it attaches itself to the mucus surface of the womb at the fundus; and as the spider weaves his web at the top of the ceiling without visible support, so by inherent power it organizes a double placenta, with a double function—one on the maternal side, and the other on the fœtal side. The double function consists of distinct tubules, innumerable in number, which ramify from both sides, without entering or being entered by either. It throws around a double decidua as a support and protection. It organizes its own blood—not a drop of the mother's blood enters it. This blood is expelled by the fœtal heart, and ramifies the fœtal side, the impurities of the blood passing by exosmosis, while those from the maternal side, by endosmosis, impart oxygen and nutrition, which is carried back to the fœtal heart, and distributed for the support and growth of itself.

Thus it will be seen that the ovaries have a single function, that of ovulation, while the womb has the incidental function of menstruation, and the real one of nursing the fœtus to maturity.

I have said that menstruation was an incidental, and not the real, function of the womb. At first, it asserts the right of an established womanhood, with all of the responsibilities and duties belonging to it. Its return after the birth of a child was doubtless to keep the parties separated for a period of four or six days, to allow the sperm to mature, the better to insure a healthy offspring. Its further significance is found in its inherent vascularity, so that, under all circumstances, whether enlarged by pregnancy and the proliferation of substance which attends it, or reduced after delivery by involution and the degeneration and absorption of adventitious substance for making new matter for the womb in pregnancy, or the absorption of it after delivery, there should be blood enough to accomplish both objects. The enlargement in pregnancy and complete involution after delivery proves conclusively that the womb was not intended to be a stationary organ. The monthly erethism and flow keeps its fibres soft and elastic by the thorough irrigation of the parenchyma, and the inner walls as well, so that its inherent functions may be performed without injury or even pain, and hence the monthly menstrual recurrence is a law to itself, in view of its real function.

Between the ovaries and womb there is but a single anatomical connection, and that is a small fibrous non-vascular ligament, which begins at the edge of each ovary and ends in an attachment to the corners of the womb at the fundus. They have no nerves, arteries or veins in common, and, from the force of circumstances, can have no function in common.

It is asserted by authors, and generally believed by medical men, that ovulation occurs monthly. This belief is founded on the doctrine of co-operative ovarian function in setting up menstruation. This cannot be true. Pregnancies occur constantly where there has been neither mollimen nor flow. I have had many such cases. I delivered one woman eight times who had never felt the mollimen or had the flow after the first pregnancy. To attempt to establish co-operation of function by a coincidence of function is certainly the wrong method of reasoning. My belief is that ovulation is not regulated by periods, but by the generative power of the organ, and that ovulation may occur once a month or once a year, and always independent of any other causation than its own inherent force. Much has been written and spoken about monthly ovulation and the washing away of the ovum by the menstrual flow. Nature never commits wholesale mistakes in this way. Every seed has its time to ripen, and then, under the proper conditions, to germinate and grow. The acorn finds its bed in the virgin soil beneath the bough, where, under the influence of heat and moisture, it may quicken and grow to the dimensions of the parent stock. So, in like manner, the ovum falls into the oviduct and finds its stroma in the womb, and, when impregnated, arrests at once, by inherent power, the mollimen and flow, and, in quiet and security, completes its great task. In view of these facts, I must think the attempt to co-relate these functions a fallacy.

I will now ask your attention very briefly to the anatomy of the womb, together with a brief summary of its actions in connection with its real and incidental functions. The womb is a cul-de-sac, with an external serous and an internal mucous surface. The virgin uterus weighs about one and three-fourths ounce. It is from one and a half to two inches in width at the fundus, tapering nearly uniformly to the neck, which is half an inch in diameter, and is from two and a half to three inches in length. The anterior and posterior body of the womb is flat. Its parenchyma consists of fibres very small and ductile, and capable of great extension, which traverse the womb longitudinally and laterally, and are held together by condensed cellular tissue, also very flexible and capable of great expansion. Its greatest thickness is about an inch; the internal surfaces almost repose upon each other. The cavity somewhat resembles the capital letter Y; the top of the Y, extending from side to side, receives the fallopian tubes at the edge of the fundus. The womb is situated at the base of the cul-de-sac of Douglass, attached posteriorly and laterally to the peritoneum, and anteriorly to the vagina. It reposes upon the rectum posteriorly, and upon the bladder anteriorly, and laterally upon the broad ligaments to which it is attached. It may change its position from side to side, or backward and forward, as the rectum and bladder happen to be full or empty, alternately. The impending omemtum and bowels may sometimes cause temporary changes in its position, and may even cause uneasiness and pain, which is quickly relieved by lying down or emptying the bowels. I have said that the womb was an inch thick, and I may add almost as solid as the same thickness and structure of gum elastic, but a little more flexible. Acute angles and flexions of the womb cannot take place except from destructive ulceration, super-involution, epithelioma, cell proliferation, or something heavy enough to hold the womb out of its axis until atrophy of the fibres takes place. The function of menstruation lasts from three to six days. The amount discharged is from five to eight ounces. It recurs every twenty-eight days. The blood discharged does not coagulate, in consequence of ammoniacal gases and other alkaline secretions, except where the flow is too great.

Regretting the necessity for this lengthy introduction, I come now to dysmenorrhœa—its pathology and treatment. The term dysmenorrhœa is derived from three Greek words, meaning a difficult monthly flow. It is very generally understood to mean painful menstruation. As normal menstruation is a painless function, when the opposite condition exists it becomes the duty of the pathologist to detect and explain as far as possible the exact nature of the departure, with all the anatomical relations to the diseased action. This is called the pathology of dysmenorrhœa. Thomas says (see "Diseases of Women:" New York; 1869; page 472): "Any condition, whether general or local, affecting the structure of the uterine walls, the ovaries, or the surrounding areolar or serous tissues, so as to render the nerves supplying these parts morbidly sensitive, may produce pain in connection with the first part of the process;" or, "anything impeding the escape of blood from the uterus or vagina may produce it by interfering with the second part." He gives, as an example, any "general condition resulting in neuralgia of the uterine or pelvic nerves, or a local inflammation altering their state, might readily create pain in the first stage, while either a natural or acquired stricture of the cervix would probably complicate the second in the same way."

He then proceeds with the subject under the following nosological arrangement, to-wit: neuralgic, congestive, inflammatory, obstructive, and membranous. He also says that it is difficult to locate the seat of pain—that "in the first varieties the pain is seated in the uterus, in the ovaries, or in the cellular tissue or peritoneum surrounding the pelvic viscera." Again he says: "Some of the most intractable cases with which I have met have been due to pelvic peritonitis or cellulitis, which, even after inflammation has subsided, has left the nerves supplying these parts in so sensitive a state that pain is excited in them by the process of menstrual congestion."—(Page 173.)

Niemeyer says it is due to flexion, nervous disorder and congestion, where there is severe pain before the commencement of the bleeding; also from mental uneasiness, which sometimes transfers the disease to remote organs, causing neuralgia and cramps, lasting sometimes to the close and even after menstruation has ceased. He says further, that these symptoms occur without perceptible organic changes, as often as otherwise, and are not confined to the plethoric, but extends to the weakly and anæmic as well; that, in some instances, the pain is caused by spasmodic contractions of the os—and again, of the uterus, like labor pains; but the symptoms do not give way until a copious hemorrhage intervenes for its relief. He thinks it difficult to determine whether the pain is kept up by hyperæmia of the pelvic organs for a long period, or by the difficulty of the escape of a graaffian follicle, deeply imbedded in the ovary and covered by thickened peritoneum. (See Niemeyer, Text-book Practical Medicine, vol. 2, pp. 143–44.)

Dr. James Y. Simpson treats the subject under two heads—ovarian and uterine dysmenorrhœa. He thinks that while generally not the seat of pain, we are justified in believing that where there is pain in the back and inguinal regions, at the period of menstruation, the ovary is probably the seat of inflammatory action, causing the pain. This opinion is founded on the existence of the kind of pain described in two females he had attended, neither of whom had even a rudimentary womb to induce the suffering of dysmenorrhœa. Allow me to say that if there was no womb the blood vessels and nerves common to it were not wanting, and there was the mollimen and stassis common to this function, but no organ to allow the escape of blood. Except as above stated he regards the uterus as the seat of the disease. He divides the disease as follows: Neuralgic, Congestive, Inflammatory, Gouty or Rheumatic, Membranous. (See Diseases of Women, page 227.

Scanzoni says, "We have already designated a good many diseases that may give rise to the phenomena of dysmenorrhœa," such as "anomalies of conformation, flexion, contractions and obliterations of the womb," etc. His special pathology is given under the head of Organic, Nervous, Congestive, Hysteralgia. Upon the authority of Gooch, he calls hysteralgia "permanent dysmenorrhœa," notwithstanding the menstrual epoch has nothing to do with the pain, which continues from day to day, month to month, and year to year. Upon the authority of Gooch again he traces the pathology of this disease to an inflamed and very painful condition of the mammary glands; thus making mastodynia a cause of the most terrible form of dysmenorrhœa, which he has not been able to cure, or even modify by remedies.

Dr. Robert Barnes (Clinical History of Diseases of Women:

London,) gives as his pathology of dysmenorrhœa the following varieties: Neuralgic or sympathetic; congestive or sympathetic; mechanical abnormalities of the uterus; fallopian obstruction; ovarian disorder constituting a distinct form.

Again, he sub-divides as follows: 1st. In which there is manifest enlargement of the uterus. 2d. Subinvolution, with chronic inflammation of the uterus following labor or abortion. 3d. Reduction of the uterus, (or flexion) most generally retroflexion. 4. A projecting conical vaginal portion, with very small os externum uteri. 5th. Lateral reclination, mostly associated with imperfect development of the uterus. 6. Disorder of distant organs, especially the digestive organs, attended or not by one or more of the preceding structural faults, and almost always by impaired sanguification and nutrition. 7th. A morbid condition of the ovaries. 8th. From various undigested and ill understood phenomena, where there is no physical fault, and from limited means of observation, he ascribes to nervous disorder.

As I wish to make the views of leading gynæcologists clear, I will refer again to Thomas, to his summing up of what seems to be the accepted doctrines of the profession on the subject of dysmenorrhœa. You will please remember that he makes five varieties, viz: neuralgic, congestive, inflammatory, obstructive and membranous. He says, in all the seat of pain is uncertain. He thinks in the first three the pain is seated in the uterus or ovaries, in the cellular tissue, or peritoneum surrounding the pelvic viscera. The worst cases he had seen were due to pelvic peritonitis or cellulitis. He does not regard dysmenorrhœa as a disease *per se*, but as a symptom of an abnormal condition, such as the neuralgic diathesis, chlorosis or plethora, certain toxemia, as malaria, gout, rheumatism, luxurious and enervating habits, together with onanism, causing the neuralgic variety. (Page 173.)

To plethora, exposure to cold, mental disturbances, sluggish portal circulation, displacement of the uterus, fibrous tumor, as the cause of congestive dysmenorrhœa. (Page 173.)

To endometritis, peri-uterine cellulitis, pelvic peritonitis, and ovaritis he ascribes inflammatory dysmenorrhœa. (Page 177.)

To contractions of the cervical canal, flexion or version of the uterus, vaginal stricture, polypus in utero, obturator hymen, or fibroid in the parenchyma of the neck he ascribes obstructive dysmenorrhœa.

He expresses no opinion of the membranous variety; some ascribing it to ovarian disease; others, as Hanfield Jones and Simpson, look upon it as an exfoliation of the uterine mucous membrane, for which no cause can be assigned. Kolb and others think it an exudation from endometritis.

I have now given the opinions in brief of five representative gynæcologists of the origin and history of this disease. I agree with them in much they say, but disagree with them in many things. I have taken great pleasure in reading and studying their views. There is an earnestness of inquiry that I admire; and a proof of the honesty with which they hold their opinions is that they are willing the world may read them and better them if they can.

I will now proceed to give my views of the etiology, pathology and treatment of dysmenorrhœa, remarking, at the same time, that I have been observing and treating this disease for near forty years, but have never written a line on the subject until now. I beg you to bear with my style, or rather the want of it.

ETIOLOGY. This disease is clearly traceable to early childhood. If the same pains were taken with children that an expert stock-raiser bestows upon his horses, etc., the result would be equally satisfactory. Comparative physiology also, if we had time to pursue the analogy, would be equally replete with valuable suggestions.

Go to Kentucky, and examine the stock farm of the late Robert A. Alexander, and ask his numerous overseers if they ever have consumption, big-head, polevil, swinny and spavin among the stock, and he will say, No. Ask him why, and he will tell you that his first care is to develop the constitution of the colt and promote its growth. Next he looks to the useful qualities. He is taught to come when called; to walk, trot or run at the word; his coat is kept clean, he sleeps warm, is fed and watered regularly, trained and educated, and it is his business to see that he is properly fed. Ask the horticulturist or gardener, and they will tell you that plants have constitutions and laws of life; while more simple than animals in their organizations yet they can no more be violated with impunity than can the laws that govern animal life.

And are children less valuable than horses, cattle and sheep, or fruit trees, vines and forests? Here is the beginning of dysmenorrhœa, and many of the most formidable diseases we are called upon to encounter besides. I wish I had time to pursue this subject, but I have not; I can only put up a finger-board here and there. Forty-nine times out of fifty dysmenorrhœa results from the want of that care that a good stock-raiser or gardener bestows upon his pets. For the rest, accidents occurring in after life, anatomical deformities and eccentricities; also, inherent or acquired vices in the constitution, etc.

PATHOLOGY. It is not necessary that I should go through the catalogue of the varieties of this disease, all of which, except my own, have been given in the copious extracts I have made from the text-books of two continents. And yet I must admit that the diseased physiology remains as much in the dark as it was forty years ago, when Bennett, the pupil of Lisfranc, gave the views of that great pathologist, as well as his own, in such glowing eloquence to the profession.

At that day, a woman now and then would seek advice generally through another person—her husband, if she had one—for dysmenorrhœa, or any other womb trouble. Dewees and Chapman were the great lights of the profession at that day. Dewees thought that the true pathology of dysmenorrhœa was due to rheumatism and gout, while his great cotemporary thought that the pathological characteristics indicated that the disease was croupal. Dr. Dewees recommended, from his standpoint, the compound tincture of guaiacum, while Dr. Chapman, from his recommended seneka snake root. When a lady patient, suffering from dysmenorrhœa, went to consult the doctor, the only thing to find out was whether it was the rheumatic or croupal form of dysmenorrhœa. That question being settled, and if rheumatic, tincture of guaiacum was given, and if no signs of rheumatism, then seneka snake root, was ordered. If a cure did not follow, a mistake in diagnosis was alleged, and the other treatment at once instituted. This, and regulating the bowels, foot and hip bathing, rest, with occasional horseback exercise, visiting the kin, and, if unmarried, matrimony, generally in a few months so far re-established the health as to be satisfactory, and there was an end of it, without mentioning the womb once. But things have changed; since Teal discoursed on tic douloureux, and Bennett on the womb, and gynæcology has become a specialty, and speculums, sponge and sea tangle tents, caustics, cutting instruments, together with several hundred varieties of pessaries and lightning rods, womb diseases have multiplied until the larger half of our women are hysterical and unhappy about some impending womb trouble that they know will kill them unless it can be cured, and they talk about "my womb" as though it were "my nose."

It is high time for thoughtful people to look this subject in the face, and redeem society from an incubus that threatens its best interests. Educate the mothers of the country up to their duty, and they will perform it with willing hands.

Many of the vices of the constitution, as well as functional ills, begin in early childhood by disturbing innervation just at the time when it most needs the sustentation of a generous hygiene, affectionate dealing with in the way of advice, and the exercise of forbearing confidence, tempered with authority. Children should not be kept too warm when awake or asleep. They should not be kept in the house when it is safe to be out of doors; nor should they be allowed to wear shoes during the thermal months until they are old enough to know when they may safely take them off. They should be taught and required to eat everything that is set before them of good quality, avoiding dainties and sweetmeats, which spoil the appetite, weaken digestion, disturb assimilation, and arrest innervation. Children should be raised pretty much as you would train a prize fighter; they eat mutton and beef as much as they will, not sodden in fats, but free from them, with a little bread, some vegetables, coffee or tea once a day, with a small amount of sugar and milk. Thus they increase the general innervation, develop muscle without fat, and elevate the constitutional forces to the highest point of activity and endurance. They should be taught to eat everything just as it should be eaten, and, more than that, compelled to do it. Every physician has lost patients because he could not nourish them, and he could not nourish them because they had been fed upon a limited diet, such as could not be resorted to when sick, and which had not made a necessary supply of lymph and blood to reinforce the system with in the emergency of a protracted illness, as typhoid fever or dysmenorrhœa, where the digestive organs are deeply involved. A lady said to me: "I don't allow my children to eat meats and coarse bread." Why, madam? "Because it makes them gross in their habits and instincts, and restless at night." It is a mistake, madam. Give your children properly cooked lean beef or mutton, fowls, etc., and teach them to eat in proper quantities whatever you put on your table; also soup, coffee, tea and milk; and when they get ill, as they will not be likely to do if thus fed, you can nourish them from some of the many articles of food they have been accustomed to eat when in health. But if they are raised on candies, nuts, tea-cakes, sugar-plums and the like, with sugar on their butter and biscuit every time they call for it, and none of the coarse meat and bread, etc., that you inveigh against, they will be miserable, unhappy beings, without health or constitutions, and when they grow to be men and women, will both be frauds upon their wives and husbands, if they ever get to be such. Feed your children thus, give them the free, open air, turn them barefooted in the warm weather, and your children will have beauty, strength and activity, and brains besides.

Many girls reach the age of puberty healthy enough; but they are going to school, and cannot be kept away because they will fall behind in their classes. They go through mud and slush, and sit with cold feet all day, with an aching back and head, while the process of menstruation is beginning, because they are too modest to speak of their peculiar illness, and too ambitious to quit school long enough to develop the function, and thus, unnoticed by mother or matron, the function becomes diseased. Every day girls are sent from home to boarding schools, where they have to ascend three or four flights of steps to their recitation rooms or dormitories, warmed by red-hot stoves, or not warmed at all, with this function just beginning or but imperfectly developed, and they are given a little hot tea at night, if anything, and told to keep up with their studies. We must exalt the functions of menstruation and ovulation to the dignity of other organs and functions, even of the brain itself, before we can cure existing evils or elevate the race to its pristine manhood and longevity, and save it from premature suffering and death. Modern civilization is crowding its innovations upon us in agonizing heaps, and they must be resisted. High-heeled shoes and boots, tight corsets with steel waists, light party dresses in midwinter that are scarcely too warm for summer, are the make-up of the modern belle. As a result, wealth and time are spent in seeking health; suffering and death follow. Out of this our profession may make money, but honor never! Our great business is to prevent disease; the lesser is to cure it.

The girl should have an ample play-ground and all the privileges of outdoor exercise consistent with the weather. She should be taught practical botany, knitting and sewing, with the minor domestic duties and pursuits, at ten years of age. At twelve she should start to school, but study no higher branches than geography and arithmetic. When the mollimen begins, or the breasts and pubes show signs of puberty, then she should be taken in hand by her mother, her bowels kept open, her feet and body kept reasonably warm, and be given free access to the open air without violent exercise; and when the mollimen begins, as it generally does, by fullness in the head, pain in the small of the back, uneasiness and bearing down in the pelvic organs, then she should have an enema of warm water, at a temperature of 103°, filling the circuit of the colon to the ilio-cæcal valve; wrap the hips in a blanket wrung out of water as hot as it can be borne, and keep this up until the menses flow as freely as necessary. If there is pain which this does not relieve, douche the womb with very warm water, thrown slowly against the portio-vaginalis, with the long tube of a Davidson syringe, for twenty or thirty minutes. If this fails, empty the warm water from the bowels, and give an enema of twenty or thirty drops of black drop, in one teaspoonful of warm water, and put this into the bowel with an ounce syringe with a long tube, say an inch at least, so as to pass it over the sphincters with certainty; but if not retained, repeat until it is, and after waiting an hour for the absorption of the black drop, then repeat the hot water enema, and continue to repeat the treatment every two hours, until a full flow is established.

The young lady, for such she now is, should still be kept from school and from hard study until two or three months shall have passed away, and the same treatment observed as directed above, until the mollimen and flow come and go without pain. If the constitution and menses are well developed together, in after life, my word for it, there will be no painful menstruation, except as the result of accidents or criminal neglect.

I come now to diseased menstruation. Beyond the fact that dysmenorrhœa is a diseased function, we are almost as much in the dark as ever. We know what will not cure, and that is perhaps something gained. The greatest gynæcological authorities of the day have not been able to locate the seat of the pain with certainty. Some ascribe it to the womb; some to the ovaries; some to the reflex forces, to inflammation of a peculiar kind, that leaves no traces behind; others again to hyperæsthesia, blood stassis, etc. Amid this poverty of facts we have appealed to fiction also. A disturbed axis comes in for its share of responsibility; notwithstanding it is demonstrable that the womb has no exact position. When the rectum is empty and the bladder is full it may be found in the trough of the sacrum; when the rectum is full and the bladder is empty it may be found encroaching anteriorly; when there is pressure from the viscera above, it dodges to one side or the other, but cannot go very far, for the reason that it is attached on either side to the broad ligaments; nor for good reasons can it be pushed downwards to any hurtful extent into the vagina, without great violence and a long train of preceding symptoms; and yet sometimes the womb does take on organic trouble requiring surgical interference. Where there is proliferation on one side and not on the other, flexion may result, and atrophy of the fibres, at the point of the greatest stress, on the weak side, take place. Or where there is ulceration of sufficient extent about the neck, a flexion will result from cicatrization; also occlusion of the inner os, more or less permanent. And again, attresia of the neck, and sometimes destruction of the entire portio-vaginalis by ulceration, or the imprudent and long continued use of caustics, causing cicatrization at the end of the vaginal cul-de-sac. But in an experience of forty years and more, I have never seen these diseases except amongst courtesans of the lowest order, or those that had been such.

The twenty-seven cases of dysmenorrhœa treated by McIntosh, twenty-five of whom were cured, and eleven bore children, is a wonderful piece of hyperbole. They are represented to have been cured by dilating the cervical canal to the size, if I recollect right, of a No. 13 bougie. Why exceed the provision of nature in this respect? There is scarcely a physician of experience that has not attempted dilatation of the cervical canal, and with what results? In my own experience, positively none. In less than half the time the experiment lasted, the canal had contracted to its original size. I have tried the smooth metallic bougie of McIntosh, sponge tents and seatangle. I have opened the portio-vaginalis from side to side, but I have never relieved sterility or cured dysmenorrhœa in a single instance, or even modified its severity for a longer period than one month, after which the same grievous sufferings returned to vex and harrow as before.

For Dr. J. Marion Sims I have the highest respect. He has shed renown upon his profession and his country. His successful treatment of vesico-vaginal and recto-vaginal diseases, his removal of cysts, tumors, etc., is a monument to his genius that will stand forever. But I have no doubt the time will come when he would be glad that half the contributions he has made to medicine and surgery, both at home and abroad, could perish forever. No man can be accurate who thinks and executes so rapidly. His treatment for sterility, by slashing the neck from side to side, by caustics, tents and the like, must fall before the light of experience. But decades must pass away before the mistakes of a great man can be corrected. An enlightened mind and benevolent heart make the great law of medicine and surgery, and when intelligent and honest convictions are followed, truth will unveil itself to the honest worshipper by and by.

It would require a volume to do justice to this subject. To point out the absurdities in pathology and treatment would be a huge task. To change the literature and the curriculum of the schools would be impossible; and existing evils will have to continue, and perhaps to multiply, until time and experience, the great revealers of truth, shall have wrought a change in our methods of inquiry and application of facts.

My treatment for dysmenorrhœa is very simple. It consists, first, in preventing the disease by establishing the function properly at the beginning—taking care, however, to resort promptly to the same means, if the disease threatens to come on again. If the patient under treatment for dysmenorrhœa is anæmic from any cause, correct the evil by an appeal to the three great functions in the nutritive processes, to-wit: digestion, absorption, and assimilation; economize all of the forces; exhaust nothing; support everything. The best general tonic for the stomach is equal parts of the tincture of quassia and the tincture of stillingia, given in drachm doses three times a day, in water. Where duodenal digestion is at fault, the means I have employed is, the tenth of a grain of the tartrate of antimony in one drachm of water, and five drops of laudanum, taken three times daily until the symptoms of duodenal dyspepsia, or the inertia of the organ, is relieved. If the lacteal system is at fault, then avoid the coarser fats and use none other than small portions of cod-liver oil, gradually increased as the patient can bear it; this, with a reasonable amount of new, sweet, faultless butter, will meet the exigencies in the lymphatic system. For making blood, the triple phosphates, where they agree with the stomach, is the best preparation I have used; also stimulants, of any kind best suited to the palate, should be used freely. Exercise in a carriage, or horseback riding, will assist materially in bringing relief to the patient. But, above all things, look to the nutritive system. If medicines, however well adapted to the disease, are found to so disagree as to lessen the capacity to take food, or interfere in any way, stop its use at once, and rally the appetite by stimulants, jellies, chicken and beef essence, exercise in the open air, etc. But when the mollimen comes on, use the hot enemas, hot blanket and douche, and also the black drop, as directed, and continue this treatment to the end of the period, and continue it at any time between the periods when there is pain, and don't allow it to exist at all.

Sometimes there are kidney troubles, not unfrequently troubles of the liver and spleen, and very often of the cord. If, however, you can reach the nutritive functions, and the patient can once begin to take food with a relish, the Rubicon is crossed. If the bowels are constipated, they should be kept regulated by giving half-grain portions of calomel, with two grains of hard loaf-sugar, carefully triturated in a mortar for ten minutes at least, and given on the tongue and mixed with the saliva, and swallowed at bed-time when the stomach is empty; and if it fails to act by morning, it should be repeated one hour before eating, and then await results. It acts mildly by restoring the secretions only, and not as a purgative. It will not impair the appetite or reduce the red matter of the blood.

Regarding dysmenorrhœa as purely a diseased function, if the nutritive organs are in good condition the only thing necessary to be done in addition is to regulate the bowels without purging them, and thereby interfering with nutritive and other supporting measures. Again, in all cases of dysmenorrhœa of the kind we are considering, the muriate of ammonia should be given three times daily. For this purpose one ounce of muriate of ammonia should be dissolved in two pints of water, and a tablespoonful given three times daily, and the use of it continued for two or three months, for the purpose of expanding the urine and defebrinating the blood, and preventing first, infarction of the arterioles and venules; and secondly, the formation of fibrous tumor, which, more frequently than many suppose to be the case, is the termination of dysmenorrhœa, and brought on by it.

Neuralgic dysmenorrhœa is often, and perhaps generally, the result of toxæmic causes, as asserted by two of the authors from whom I have quoted. But it may arise from other causes also; as congestion, commencement of malignant disease, hyperæsthesia of a part, or all, of the sensory uterine nerves. But no matter what may be the cause, the treatment I have laid down will give perfect relief, until other remedies can be brought to bear, such as arsenic, quinine, iron, cicuta, etc.

In membranous dysmenorrhœa I advise the hot water and black drop treatment, and also mild ptyalism, muriate of ammonia daily for three months, and keeping the bowels scrupulously regulated, but not purged; for this purpose the compound cathartic pill is the best. If there be whites, as there generally is, I make a wash of two ounces muriated tincture of iron and four of water; and every night I elevate the hips on a pillow, and throw half an ounce as far as I can carry a glass female syringe into the vagina and discharge it. This I repeat every night, and, in severe cases, three times daily for two or three days, and afterwards every one or two days repeat at night, until there is a discharge of loose, diseased epithelium, organized by the wash into a skinny substance resembling yellowish paper, which passes in strips as large sometimes as the hand, and sometimes the complete cast of the vagina will come at once. When these slips begin to come, encourage them to do so by the douche, but continue the iron until this ceases, or the part becomes too much inflamed to go further. Whenever the whites show a disposition to return, do the same thing over again, and continue until the symptom yields.

Scanzoni says, on the authority of Gooch, that the form of dysmenorrhœa known as hysteralgia is due to mastodynia; and while I do not believe its pathology traceable to that cause, the fact remains that the breasts are very much swollen and sore a large portion of the time. I am treating a case now that I believe will recover, unless something malignant developes. The mollimen is violent and the mammary glands exceedingly sore for ten days before the flow begins. When it comes on, it is very free, but does not bring relief. It lasts about six days—sometimes longer. When it passes away, the pain in the womb remains, and returns once or twice daily. The pain and swelling of the breasts, however, cease gradually after the flow is over. I have been using the hot water and black drop, always with present relief, but the symptoms recur daily. She cannot take the muriate of ammonia, and I have determined to try the carbonate, and have thus prescribed, but do not know with what success up to this moment. I base my hopes of success on resisting the pain, overcoming congestion, elimination of fibrine by ammonia, and restoration by nutrition and hygiene.

Lastly, obstructive dysmenorrhœa calls for a few remarks. Thomas ascribes this variety to contractions of the cervical canal, flexion or version of the uterus, vaginal stricture, polypus-in-utero, obturator hymen, or fibroid in the parenchyma of the neck. I treat this just as I have recommended for the other varieties of this disease. They are, in fact, one disease, with diverging anomalies. We have to deal with a diseased function. My object is to call attention to this fact, and also to a treatment in harmony with it. By carefully soothing the diseased organ in the manner suggested, you will rob dysmenorrhœa of its horrors. By strict attention to the nutritive system, by proper exercise in the open air, restraint from excesses, going early to bed and sleeping well, the process of restoration will be inaugurated, and great results follow. By this treatment I have cured two cases of hysteralgia, pronounced incurable by leading gynæcologists; also, three of membranous dysmenorrhœa (all I ever treated); also, metritis and perimetritis; and, if taken in time, cellulitis will disappear from the catalogue of pelvic diseases. Leucorrhœa is nearly an invariable attendant upon dysmenorrhœa. This can be certainly cured by using, as a vaginal enema, the muriated tincture of iron, one-third or one-half strength, every night and morning, introduced with a blunt female syringe—half an ounce of the mixture to be thrown down to the base of the vaginal cul-de-sac, and the portio-vaginalis compelled to remain in the bath all night, by placing a pillow under the hips; the fluid will then penetrate the cervical canal to the os; and, by keeping this up for three nights, until the discharge ceases, you will have cured the disease. If it returns, treat it the same way.

With the treatment for dysmenorrhœa heretofore recommended and the muriated tincture of iron, used as above, I have cured the most inveterate cases of uterine catarrh. Uterine surgery should only be resorted to after everything else has been tried; and if the treatment here proposed be followed out, and defibrinating remedies properly and persistently used, in my opinion there will not be much of it required.

And finally, keep it before you that dysmenorrhœa developes a majority of the diseases that women suffer from. Their name is legion. But, bring on the menses properly, keep the function healthy, treat the diseased function as indicated, pay special attention to the nutritive organs, regulate the bowels well to one action a day, and you will cure a vast majority, and make life a joy and not a burden to all the unfortunate victims to dysmenorrhœa.

